# Summary of the best evidence for the use of antiseptics at various surgical sites to prevent postoperative infections

**DOI:** 10.3389/fmed.2025.1630272

**Published:** 2025-08-08

**Authors:** Qingqing Du, Shan Wu, Ziwei Jin, Li Ni

**Affiliations:** ^1^Tongji University School of Medicine, Shanghai, China; ^2^Shanghai Children’s Hospital, School of Medicine, Shanghai Jiao Tong University, Shanghai, China; ^3^Shanghai East Hospital, School of Medicine, Tongji University, Shanghai, China

**Keywords:** surgical site infection, antiseptics, evidence-based nursing, best evidence, systematic review

## Abstract

**Objective:**

To retrieve and summarize the best available evidence regarding the use of antiseptics at various surgical sites to prevent postoperative infections.

**Methods:**

Following the “6S” evidence model, a comprehensive search was conducted across guideline repositories, professional association websites, and both Chinese and English databases. The search covered literature from database inception through December 2024. Two researchers trained in evidence-based nursing independently screened the literature, assessed quality, extracted data, and synthesized the findings.

**Results:**

A total of 30 studies met the inclusion criteria, consisting of 3 clinical decision support documents, 5 guidelines, 4 expert consensuses, 9 systematic reviews, and 9 evidence summaries. In total, 36 pieces of evidence were integrated across five key areas: general principles, recommended antiseptics for specific surgical sites, application methods, handling of special circumstances, and quality control.

**Conclusion:**

This study compiles the best current evidence on antiseptic use across different surgical sites for preventing postoperative infections. It lays a foundation for standardizing disinfection protocols and improving infection control in clinical practice. Healthcare professionals are encouraged to integrate this evidence with individual patient conditions and clinical judgment.

## 1 Introduction

Surgical site infection (SSI) remains one of the most frequent hospital-acquired infections. It not only extends hospital stays and drives up healthcare costs, but can also cause serious complications or even result in death (1–3). Reports indicate that the incidence of SSI is between 1 and 5% in clean surgeries, while in contaminated operations, it may increase to 20–40% (2–4). Among the various preventive approaches, selecting and applying antiseptics appropriately is considered a critical measure (1, 2, 5). Antiseptics commonly used in clinical settings include povidone-iodine, alcohol-based solutions, chlorhexidine, and combinations of these agents (6–8). Despite their widespread use, a unified protocol for choosing and applying antiseptics across different surgical sites has yet to be established. This study aimed to systematically search and synthesize relevant domestic and international literature to identify the best available evidence, offering evidence-based recommendations for clinical use.

## 2 Materials and methods

### 2.1 Formulation of the research question

The evidence-based clinical question was structured using the PIPOST framework (4), which comprises six key elements:

- Population (P): Patients undergoing surgical procedures;
- Intervention (I): Selection and application of antiseptics at surgical sites;
- Professionals (P): Operating room healthcare teams;
- Outcomes (O): Surgical Site Infection (SSI) rates, antiseptic efficacy, and adverse reactions;
- Setting (S): Hospital operating theaters;
- Type of evidence (T): Clinical decision-making tools, guidelines, and systematic reviews.

### 2.2 Literature search strategy

Using the “6S” evidence hierarchy model, a top-down approach was adopted to retrieve relevant literature (3, 9). The databases and platforms searched included UpToDate, BMJ Best Practice, Cochrane Library, JBI Evidence-Based Healthcare Database, National Guideline Clearinghouse (NGC), YiMaiTong, EBSCOhost, PubMed, Web of Science, JAMA, Agency for Healthcare Research and Quality (AHRQ), along with CNKI, Wanfang Data, SinoMed, VIP Database, Chinese Medical Association, *The New England Journal of Medicine*, *The Lancet*, *Chinese Medical Journal*, *Chinese Journal of Surgery*, and *Chinese Journal of Practical Surgery*.

Corresponding search terms included combinations of: “surgical site infection/SSI/postoperative infection/complications/perioperative care/preoperative skin preparation/surgical wound/incision/operating room/theatre/aseptic technique/sterile technique” “antiseptic/antisepsis/disinfectant/disinfection/skin preparation solution/surgical prep solution/chlorhexidine gluconate/CHG/povidone-iodine/PVP-I/alcohol-based antiseptic/octenidine/surgical scrub/antimicrobial agent” “prevention/preventive measures/infection control/risk reduction/evidence-based practice/clinical practice guidelines/best practice/quality improvement/patient safety.” The search covered all records from database inception to December 2024.

### 2.3 Inclusion and exclusion criteria

Inclusion criteria: (1) Studies involving surgical patients; (2) Research focused on the selection and application of surgical site antiseptics; (3) Publications available in Chinese or English. Exclusion criteria: (1) Studies with incomplete data or duplicate entries; (2) Articles without accessible full texts; (3) Outdated studies that had been replaced by updated versions; (4) Studies with poor methodological quality.

### 2.4 Literature quality assessment

1. Articles from UpToDate and BMJ Best Practice, positioned at the top of the evidence hierarchy, were directly included due to their high level of evidence.
2. Guidelines were assessed using the AGREE II instrument, which includes 23 items across 6 domains (3).
3. Expert consensus documents were appraised according to the JBI critical appraisal criteria.
4. Systematic reviews were evaluated using the AMSTAR-2 tool (7, 10).
5. Evidence summaries were assessed based on the quality of the original studies they referenced.

All literature screening and evaluations were independently performed by two trained researchers. Any disagreements were resolved through discussion with a third reviewer.

### 2.5 Evidence extraction and synthesis

Evidence extraction was independently conducted by two researchers trained in evidence-based nursing. In cases where opinions differed, a third researcher joined the discussion to reach agreement. The process emphasized high-quality, evidence-based, and authoritative sources.

### 2.6 Evidence grading

The final evidence was graded following the recommendation system established by the Joanna Briggs Institute (JBI), Australia (2016 edition). Level 1 = Meta analysis of homogeneous RCT; Level 2 = high quality RCT; Level 3 = quasi-experimental study; Level 4 = observational study; Level 5 = expert opinion. When the same recommendation contains multiple levels of evidence, the highest level is indicated and the source of differences is explained (3). Depending on differences in study design, the original studies contributing to the best evidence were classified into levels 1 through 5.

## 3 Results

### 3.1 Literature search results

**Figure 1:**
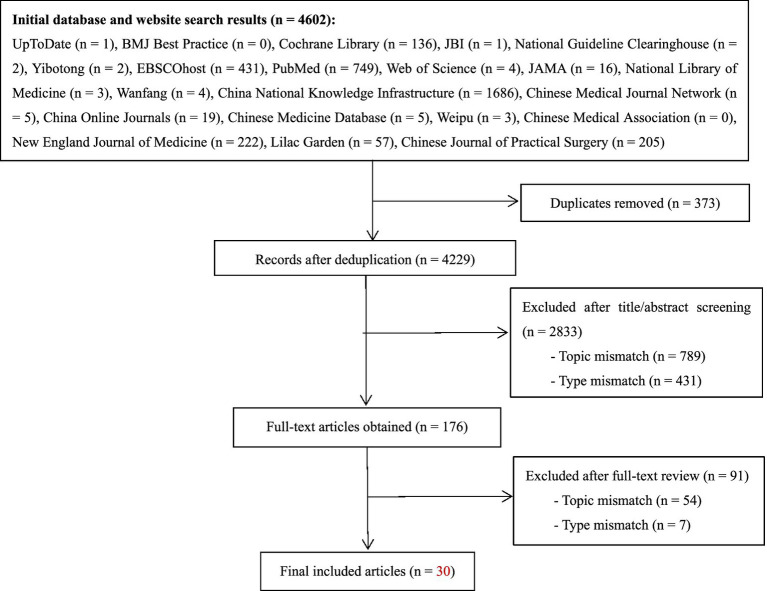
Flowchart of literature screening.

A total of 4,602 records were identified. After removing duplicates and conducting an initial screening, 98 articles remained. Following title and abstract review, 45 articles were selected for full-text assessment. Ultimately, 30 articles were included: 3 clinical decision summaries, 5 guidelines, 4 expert consensus statements, 9 systematic reviews, and 9 evidence summaries. The screening flow is shown in Figure 1, and the basic characteristics of the included studies are detailed in Table 1.

**Table 1:** Basic characteristics of included studies.

First author	Year	Study topic	Type of literature	Source journal/publisher
Andersen B (1)	2018	Control of Surgical Site Infections	Clinical Decision Support	Prevention and Control of Infections in Hospitals
Berríos-Torres S (2)	2017	Guidelines for the Prevention of Surgical Site Infections	Clinical Decision Support	JAMA Surgery
Bratzler D (20)	2013	Guidelines for Surgical Antimicrobial Prophylaxis	Clinical Decision Support	Surgical Infections
WHO Guidelines Development Group (3)	2016	Global Guidelines on the Prevention of Surgical Site Infections	Guideline	World Health Organization
NICE Guideline Development Group (9)	2020	Prevention and Treatment of Surgical Site Infections	Guideline	National Institute for Health and Care Excellence
Chinese Society of Surgical Infection and Intensive Medicine (4)	2019	Guidelines for the Prevention of Surgical Site Infections	Guideline	Chinese Journal of Gastrointestinal Surgery
Allegranzi B (5)	2016	Recommendations for Preoperative Infection Prevention Measures	Guideline	The Lancet Infectious Diseases
AORN (6)	2022	Guidelines for Preoperative Skin Antisepsis	Guideline	AORN Guidelines for Perioperative Practice
Chinese Society of Hospital Infection (11)	2020	Prevention and Control of Surgical Site Infections	Expert Consensus	Chinese Journal of Hospital Infection
Mao Yanjun (21)	2022	Infection Prevention in Interventional Operating Rooms	Expert Consensus	Journal of Interventional Radiology
Gómez-Barrena E (13)	2022	Prevention of Periprosthetic Joint Infections	Expert Consensus	Journal of Clinical Medicine
Munoz-Price L (22)	2018	Infection Prevention in Anesthesia Zones of Operating Rooms	Expert Consensus	Infection Control & Hospital Epidemiology
Dumville J (7)	2015	Preoperative Skin Antisepsis for Clean Surgeries	Systematic Review	Cochrane Database of Systematic Reviews
Mastrocola M (10)	2021	Comparison of Preoperative Skin Antisepsis Protocols in Orthopedic Surgeries	Systematic Review	Scientific Reports
Lee I (8)	2010	Comparison of Chlorhexidine and Povidone-Iodine for Preoperative Antisepsis	Systematic Review	Infection Control & Hospital Epidemiology
Noorani A (23)	2010	Comparison of Antisepsis Protocols for Clean-Contaminated Surgeries	Systematic Review	British Journal of Surgery
Liu Jian (17)	2021	Comparison of Two Antiseptics in Infection Prevention	Systematic Review	Nursing Research
Jiang Xuesong (18)	2013	Efficacy of Skin Antiseptics in Infection Prevention	Systematic Review	Chinese Journal of Hospital Infection
Anggrahita T (24)	2017	Efficacy of Compound Antiseptics	Systematic Review	Medical Journal of Indonesia
Wood TJ (25)	2020	Selection of Irrigation Solutions for Joint Replacement	Systematic Review	Cureus
Fu Zhongmin (26)	2022	Evidence-Based Prevention of Infection in Joint Replacement Surgeries	Evidence Summary	Chinese Journal of Infection Control
Zhao Wenting (27)	2023	Preoperative Ocular Antisepsis in Cataract Surgery	Evidence Summary	Tianjin Nursing
Meoli A (12)	2022	Infection Prevention Measures in Pediatric Surgeries	Evidence Summary	Antibiotics
Liu Yulin (28)	2023	Infection Prevention and Control in Cataract Surgery	Evidence Summary	Shanghai Nursing
Zhang Yizhi (29)	2023	Pre-cleaning Protocols for Surgical Instruments	Evidence Summary	Nurse Training Journal
Giamarellou H (15)	2023	Antimicrobial Stewardship in the Hospital Setting	Evidence Summary	Antibiotics
Meena R (14)	2023	Clinical evaluation of preoperative skin preparation with aqueous povidone iodine only and in combination with alcoholic chlorhexidine in patients undergoing clean elective surgeries	Systematic Review	Journal of Clinical Images and Medical Case Reports
Ziogou A (30)	2023	Post caesarian section surgical site infections	Evidence Summary	Hellenic Journal of Obstetrics and Gynecology
Borgia A (19)	2023	Prophylaxis of Ocular Infection in the Setting of Intraocular Surgery: Implications for Clinical Practice and Risk Management	Evidence Summary	Ophthalmology and Therapy
Lei Qingmei (16)	2024	Evidence-based analysis for surgical site infection prevention in adult inpatients based on guidelines and clinical decision-making	Evidence Summary	Modern Hospitals

### 3.2 Literature quality evaluation results

#### 3.2.1 Guidelines

Five surgical site disinfection guidelines were assessed using the AGREE II tool. The WHO guideline received the highest scores across all domains (ranging from 85 to 95%), followed by the CDC guideline (85–93%), the NICE guideline (83–90%), and the AORN guideline (80–88%). The Chinese Medical Association guideline had relatively lower scores in all areas (78–85%). Higher scores were observed in domains such as scope and purpose, editorial independence, and rigor of development, whereas the applicability domain consistently scored lower. Based on the evaluations, the WHO, CDC, NICE, and AORN guidelines were recommended for use, while the Chinese Medical Association guideline was recommended with reservations.

#### 3.2.2 Systematic reviews (AMSTAR-2 score)

Of the 8 systematic reviews assessed with the AMSTAR-2 tool, 3 were classified as high quality, 4 as moderate quality, and 1 low-quality review was excluded. The compliance rates across various evaluation items differed: all reviews met the criteria for formulating research questions and inclusion criteria, as well as conducting comprehensive literature searches, yielding a 100% compliance rate. Literature selection methods, screening processes, and the reporting of included study details each showed an 87.5% compliance rate. Registration of study protocols and data extraction processes had a 75% compliance rate. Reporting of excluded studies was weaker, with a compliance rate of 62.5%. These results suggest that while the overall methodological quality of the included systematic reviews was sound, reporting on excluded studies requires further attention.

#### 3.2.3 Expert consensus

All four expert consensus documents adopted the Delphi method for gathering expert opinions, each undergoing 3 to 4 rounds of consultation. The expert panels were composed of professionals from surgery, infection control, nursing, and related fields, with a well-balanced mix of expertise. Agreement among experts exceeded 80%.

#### 3.2.4 Randomized controlled trials

All randomized controlled trials included in the study achieved a Jadad scale score of 3 or above, reflecting solid methodological quality. Each study employed appropriate randomization, incorporated a double-blind design, fully reported participant dropout and loss to follow-up, and maintained adequate follow-up periods. These aspects indicate that the design and execution of the trials were of high quality, and the findings are considered reliable.

### 3.3 Evidence summary

The evidence drawn from the included literature was synthesized into a set of best evidence for the use of antiseptics across different surgical sites to prevent postoperative infections. This covered five domains: general principles (7 items), recommended antiseptic agents for surgical site (12 items), application methods (8 items), management of special circumstances (5 items), and quality control (4 items), with a total of 36 evidence statements (see Table 2).

**Table 2:** Summary of best evidence for the use of antiseptic at different surgical sites to prevent postoperative infections.

Category	No.	Evidence description	Evidence level
General principles	1	Preoperative assessment should include the type of surgery, site characteristics, individual patient conditions, and allergy history (1–3).	Level 2
	2	Selection should follow principles of efficacy, safety, and cost-effectiveness, taking into account patient-specific factors (3, 6, 11).	Level 5
	3	Strict adherence to sterile techniques to ensure full coverage of the surgical area and surrounding skin (2, 3, 5).	Level 1
	4	Disinfection should follow the basic principle of “inside-out, top-down, clean to contaminated” (6, 11).	Level 5
	5	Prepare according to the manufacturer’s instructions, using fresh solutions with proper concentration (3, 6).	Level 5
	6	Antiseptics should be sealed and stored properly to avoid contamination and degradation during their shelf life (6, 11).	Level 5
	7	Routine evaluation and monitoring of surgical site disinfection effectiveness should be conducted (3, 5).	Level 2
Selection of antiseptic	8	The preferred disinfectant for clean surgeries is a 2% chlorhexidine-alcohol + 75% alcohol compound, which can reduce the incidence of surgical site infections (SSIs) (7, 8, 10, 24).	Level 1
	9	Iodine-based antiseptics are recommended, with caution to prevent liquid from entering eyes, ears, or nasal cavities (3, 6, 11).	Level 2
	10	Use diluted iodine solution (0.5–1%) and avoid alcohol-based antiseptic (3, 11, 30).	Level 2
	11	The preferred disinfectant is a 2% chlorhexidine-alcohol compound, with extended disinfection time of 3–5 min before surgery (10, 13, 25, 26).	Level 1
	12	Use a 2% chlorhexidine-alcohol + 75% alcohol compound, ensuring that the liquid does not pool and cause skin damage (3, 6, 8).	Level 2
	13	Recommended antiseptic include a 2% chlorhexidine-alcohol compound or iodine, with the disinfection area extending 5–10 cm beyond the incision (8, 17, 23).	Level 1
	14	Iodine or chlorhexidine-alcohol solutions may be used, with particular attention to spaces between fingers and toes (3, 6, 11).	Level 2
	15	Diluted iodine (0.5–1%) is preferred, with care to avoid irritating mucosal surfaces (3, 11, 30).	Level 2
	16	Transparent, colorless antiseptic are preferred to easily observe skin reactions (6, 11).	Level 5
	17	Quick-drying formulations should be used at puncture sites to prevent seepage into the incision (14, 21).	Level 2
	18	In pediatric surgery, low-concentration antiseptics 1% chlorhexidine (non-alcoholic formulation) should be used to minimize skin irritation, taking age-related differences into account (12).	Level 2
	19	Gentle antiseptic should be used to avoid worsening tissue damage (3, 24).	Level 2
Application methods	20	Ensure uniform mixing of components before use (3, 6).	Level 5
	21	Apply the antiseptic in a spiral motion from inside out, repeating 2–3 times with 30-s intervals, for a total contact time of 2–3 min (7, 8, 10).	Level 1
	22	Repeat application 3–5 times, waiting for the color to deepen before reapplying, with a total contact time of 3–5 min (8, 23).	Level 1
	23	Each layer must be allowed to dry completely before the next is applied to avoid dilution (3, 6, 8).	Level 2
	24	The disinfection area should extend 10–15 cm beyond the surgical region to ensure a sterile field (3, 11).	Level 2
	25	Skin folds should be spread adequately to ensure even coverage of the disinfectant (6, 11).	Level 5
	26	Use sterile cotton swabs or wipes for each application to prevent cross-contamination (3, 6).	Level 2
	27	Allow the disinfectant to dry fully before applying sterile drapes, to avoid penetration of the disinfectant (3, 6, 11).	Level 2
Special situations	28	Patients with allergies must be carefully evaluated and undergo skin testing to determine a suitable antiseptic alternative (3, 14).	Level 2
	29	For infected wounds, compound antiseptics should be used with longer application time and a wider coverage area (3, 15).	Level 3
	30	Patients with immunodeficiency should opt for broad-spectrum, long-acting compound preparations, extend the disinfection time to 5 min, and combine barrier protection to enhance the disinfection effect (15, 16, 19, 30).	Level 2
	31	For patients with skin lesions, gentle antiseptic should be selected to avoid exacerbating skin damage (3, 11).	Level 3
	32	For patients undergoing repeated surgeries, skin tolerance should be assessed before antiseptic selection to avoid adverse skin reactions (3, 11, 16).	Level 4
Quality control	33	Implement a system for monitoring surgical site infections, with regular collection and review of infection data (3, 5, 15).	Level 2
	34	Maintain accurate records for antiseptic use, including batch numbers, expiry dates, and preparation times (3, 6).	Level 5
	35	Conduct regular assessments of disinfection outcomes, including bacterial cultures and resistance tracking (3, 5, 15).	Level 2
	36	Establish a reporting mechanism for adverse reactions to antiseptics, with timely identification and management of complications (3, 6, 11).	Level 5

## 4 Discussion

### 4.1 Standardized disinfection as the key to preventing surgical site infections

Surgical site infections, a major category of hospital-acquired infections, are closely linked to how well disinfection procedures are designed and followed. Based on a comprehensive literature review and evidence assessment, this study presents a multifaceted prevention system built on 36 pieces of best evidence. The results show that because surgical sites differ in anatomical structure and physiological traits, the selection of antiseptics and the protocols for their application must also differ. Clinicians must consider multiple factors—such as the type of surgery, the characteristics of the surgical site, and the patient’s individual condition—when designing disinfection strategies. This tailored approach supports both accuracy and patient-specific care in clinical disinfection (1–3, 5).

This study integrates evidence from five major sources (clinical decision-making, guidelines, consensus, systematic reviews, and evidence synthesis) to construct a cross-departmental decision framework. By synthesizing surgical site anatomical characteristics, patient individual differences, and evidence-based data on disinfectants, it addresses practical gaps in existing guidelines for specialized scenarios such as ophthalmic procedures, pediatric mucosal surgeries, and immunosuppressed patients. The research provides interdisciplinary support for establishing the “Standards for Surgical Disinfectant Application” (4, 11, 12).

### 4.2 The importance of personalized disinfection protocols

The principle of individualized care has become a central theme in modern medical practice. This study stresses that the personalization of disinfection strategies is essential—not only for patients with specific needs, such as those with allergies or weakened immune systems, but also in accounting for the distinct nature of different surgical sites. Allergic reactions to antiseptics are a notable clinical issue that must not be overlooked. Table 3 outlines common types of antiseptic allergies, their clinical manifestations, and appropriate management approaches.

**Table 3:** Guide to disinfectant selection for surgical sites.

Surgical site/type	Preferred disinfectant	Alternative scheme	Contraindications/precautions	Evidence level
Clean surgical (routine skin)	2% Chlorhexidine gluconate alcohol solution	Iodophor solution	Avoid contact with eyes, ears, and nasal cavity	Level 1
Mucous membranes/Eye, Ear, Nose surgery	0.5–1% Diluted iodophor solution	Benzalkonium chloride solution	Alcohol/Chlorhexidine contraindicated	Level 2
Orthopedic/Joint replacement	2% Chlorhexidine-alcohol (apply for 3–5 min)	Iodophor-alcohol combination	Ensure disinfectant does not pool in skin folds	Level 1
Hand/Foot surgery	Chlorhexidine-alcohol or Iodophor		Focus on disinfecting finger/toe webs	Level 2
Pediatric surgery	0.5% Chlorhexidine or diluted iodophor	Benzalkonium chloride	Avoid high-concentration agents; monitor skin tolerance	Level 2
Immunocompromised patients	Broad-spectrum combination disinfectant (extended contact time)		Extend disinfection time to ≥5 min	Level 2

The incidence of SSI in newborns and children ranges from 0.18 to 6.8%, primarily depending on the patient’s age and surgical type. Standardized sterilization protocols widely used in adult surgeries may prove ineffective or even harmful in pediatric populations due to physiological and pathogen-specific characteristics. First, pediatric surgeries should consider age-related factors by using low-concentration antiseptics to minimize skin irritation. Second, neonatal and pediatric SSI pathogens exhibit distinct patterns: coagulase-negative staphylococci and methicillin-resistant *Staphylococcus aureus* are predominant in newborns, while *Escherichia coli* and Candida species dominate post-intestinal surgery in children. Disinfectant selection should therefore be tailored to specific surgical procedures. Additionally, individualized oxygen management is crucial. Premature infants require strict SpO_2_ control (88–94%) to prevent retinopathy of the retina (ROP), whereas full-term infants can tolerate higher oxygen levels to reduce SSI risks. Implementing personalized disinfection strategies remains the only way to balance safety and effectiveness in pediatric care (6, 12).

Every patient presents with individual differences, and factors such as skin integrity, underlying conditions, and prior surgical history can all influence disinfection outcomes. An effective disinfection strategy should be grounded in a thorough assessment of the patient’s overall status. Tailored interventions allow for more precise prevention, ensuring that disinfection procedures align with each patient’s specific needs (3, 12–14).

### 4.3 Quality control and continuous improvement

Building a robust quality control system is central to ensuring effective disinfection. This requires the integration of several key components: developing a scientific surgical site infection surveillance system, standardizing the entire process of antiseptic use, performing regular assessments of disinfection outcomes, and establishing a fast-response mechanism for reporting and managing adverse reactions. Importantly, ongoing education and practical training for healthcare staff are essential. Through repeated assessments of practical skills and standardized operating training, clinical personnel can maintain a high level of competence, ensuring that disinfection quality is upheld by capable, well-prepared teams (3, 5, 15, 16).

### 4.4 Emerging disinfection technologies and cost–benefit analysis

**Figure 2:**
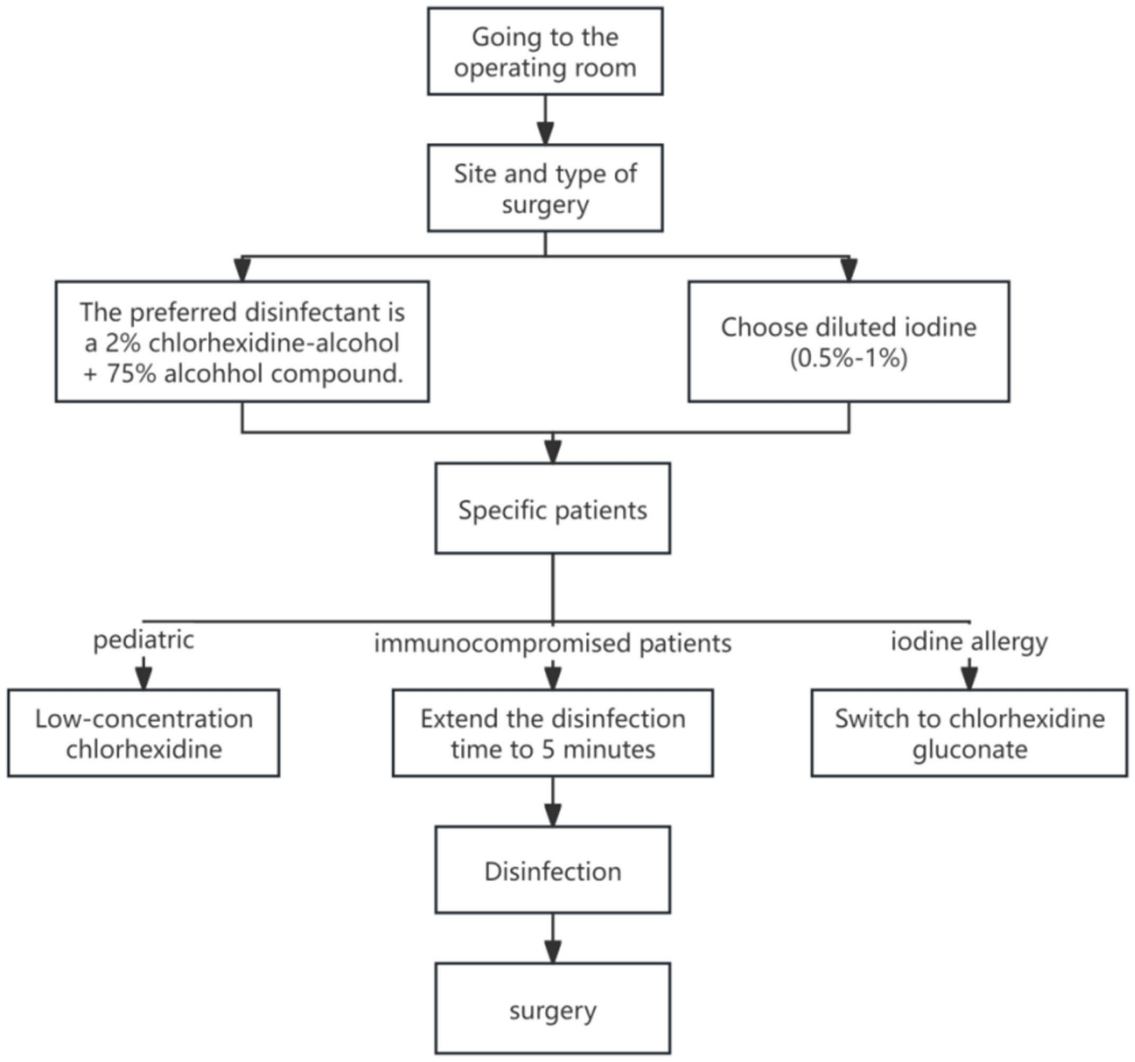
Clinical decision flow chart for surgical disinfectants (based on evidence 8, 9, 10, 18, 28).

In real-world practice, evidence-based measures for preventing surgical site infections (SSI) often face implementation challenges due to cost constraints, resource availability, and patient hypersensitivity. Taking preoperative skin disinfection as an example: while studies (17, 18) confirm that CHG ethanol solution outperforms povidone-iodine in reducing SSI incidence, its higher costs hinder widespread adoption. Additionally, patient allergies to certain disinfectants (3, 14) may force clinicians to adopt suboptimal alternatives, potentially diminishing preventive efficacy. In resource-limited regions or primary care facilities, disposable sterile surgical packs and antimicrobial-coated sutures remain impractical due to high costs, while reusable cotton surgical packs increase infection risks. For vulnerable populations like newborns and children with compromised skin barriers, the use of low-concentration formulations such as 1% chlorhexidine (non-alcoholic formulation) (12) further restricts the applicability of highly evidence-based protocols. These practical contradictions highlight the need for dynamic adjustments to SSI prevention strategies based on institutional budgets, patient characteristics, and allergy histories, rather than rigidly applying guidelines (Figure 2; Tables 4–6).

**Table 4:** Guide to disinfectant selection by surgical scenario classification.

Disinfectant type	Clean surgery	Clean-Contaminated surgery	Contaminated surgery	Mucous membrane surgery	Contraindications/precautions	Evidence level
Chlorhexidine-alcohol solution	Preferred	Preferred	Optional	Not recommended	Long residual time, broad-spectrum, not for mucous membranes	Level 1
Povidone-iodine (PVP-I)	Alternative	Alternative	Optional	Preferred	Rapid onset, not suitable for mucous membranes or open wounds	Level 2
Diluted iodophor solution	Not recommended	Can be used for irrigation	Can be used for irrigation	Optional	Can be used on mucous membranes or intraoperative irrigation; moderate efficacy	Level 2
Low-concentration chlorhexidine	Not recommended	Not recommended	Not recommended	Optional	Can be used on mucous membranes; weak efficacy, short residual time	Level 2
Hydrogen peroxide	Not recommended	Not recommended	Can be used for irrigation	Not recommended	Only for adjunctive irrigation of contaminated wounds	Level 2
Benzalkonium chloride (BZK)	Not recommended	Not recommended	Can be used for irrigation	Not recommended	Suitable for adjunctive cleaning of mildly contaminated skin	Level 2

**Table 5:** Comparison of disinfectant properties.

Disinfectant	Suitable sites	Contraindicated sites	Contact time	Evidence level
2% Chlorhexidine-alcohol	Skin/Clean incisions	Cochlea/Cornea	≥2 min	Level 1^[13,15]^
0.5–1% Iodophor	Mucous membranes/Contaminated incisions	Thyroid surgery	≥3 min	Level 2^[4,28]^
70–90% Alcohol	Intact skin	Wounds/Neonates	30 s	Level 3^[8,23]^
Benzalkonium chloride (BZK)	Ophthalmic/Urological	Open wounds	≥1 min	Level 4^[22,24]^

**Table 6:** Comparison of disinfectant allergy types.

Allergy type	Clinical manifestations	Emergency management	Alternative
Iodine allergy	Erythema, urticaria, dyspnea	Discontinue use → Saline irrigation → Epinephrine injection	Chlorhexidine (non-iodine), BZK
Chlorhexidine allergy	Contact dermatitis, angioedema	Antihistamines + Corticosteroids	Iodophor, Alcohol-only solution
Alcohol allergy	Skin fissures, burning pain	Cover with petroleum jelly for moisturization	Aqueous iodophor, Aqueous chlorhexidine

Emerging technologies like sustained-release chlorhexidine dressings (15) and photodynamic disinfection (19) show promise in orthopedic implant surgeries. Animal studies demonstrate that nanosilver dressings can reduce SSI risk by 40% (RR = 0.60) (15), though their mucosal bioavailability requires further clinical validation. Cost analysis reveals that chlorhexidine-alcohol formulations are 2.3 times more expensive per unit than iodine tincture, yet their reduced SSI incidence could save total healthcare expenditures by (8). It is recommended that institutions with limited resources prioritize high-risk procedures (joint replacement/heart surgery).

### 4.5 Limitations of this study

While this study offers a relatively comprehensive, evidence-based framework, several limitations remain. Language constraints may have led to the exclusion of high-quality studies not published in Chinese or English. Some included evidence is based on expert consensus, which carries a lower level of credibility compared to empirical studies. Moreover, practical application in clinical settings requires consideration of real-world conditions, such as institutional resources and cost-effectiveness (15, 16, 19). Future research should address the following areas: (1) conduct more high-quality randomized controlled trials, especially on personalized disinfection strategies for specific patient groups; (2) explore the potential of new disinfectant materials and technologies in preventing surgical site infections; (3) develop a more complete evaluation system for disinfection outcomes, incorporating economic indicators into effectiveness assessments.

## 5 Conclusion

This study systematically reviewed and compiled the best available evidence on the use of antiseptics to prevent surgical site infections. The findings offer a solid theoretical basis for clinical application and practical guidance for institutions in formulating individualized prevention strategies. Healthcare professionals are encouraged to design disinfection protocols that are both scientifically sound and tailored to available resources and patient-specific factors. In addition, the establishment of a complete quality control framework and ongoing improvement mechanisms will help strengthen infection prevention efforts, contributing to better patient safety and overall healthcare quality.

These findings lay an important foundation for both the theory and practice of surgical site infection control. Still, successful clinical application requires continuous observation and sound clinical judgment. While adhering to evidence-based recommendations, clinicians must also account for individual differences to ensure targeted and effective prevention. Future research should expand on current evidence and examine the role of emerging technologies and methods in this area, supporting further progress and innovation in surgical site infection prevention.

## Data Availability

The raw data supporting the conclusions of this article will be made available by the authors, without undue reservation.
