# Correction: Summary of the best evidence for the use of antiseptics at various surgical sites to prevent postoperative infections

**DOI:** 10.3389/fmed.2025.1698325

**Published:** 2025-09-05

**Authors:** Frontiers Production Office

**Affiliations:** Frontiers Media SA, Lausanne, Switzerland

**Keywords:** surgical site infection, antiseptics, evidence-based nursing, best evidence, systematic review

There was a discrepancy in the affiliations and funding statement between the PDF and HTML/XML versions of the paper. In the PDF, affiliation 1 was given as “Shanghai Children's Hospital, School of Medicine, Shanghai Jiao Tong University, Shanghai, China” and affiliation 2 was given as “Tongji University School of Medicine, Shanghai, China”. The correct affiliations are “1 Tongji University School of Medicine, Shanghai, China” and “2 Shanghai Children's Hospital, School of Medicine, Shanghai Jiao Tong University, Shanghai, China”.

In addition, the Funding statement in the PDF was erroneously given as “The author(s) declare that financial support was received for the research and/or publication of this article. This work was supported by Hospital-Level Nursing Special Research Project of Shanghai Children's Hospital in 2023 (grant no: 2024HLZX05).”

The correct Funding statement reads:

“The author(s) declare that financial support was received for the research and/or publication of this article. This work was funded by Academic Leaders Training Program of Pudong Health Bureau of Shanghai (grant no. PWRd2021-19).”

The affiliations and Funding statement were correct in the HTML and XML versions of the paper at time of publication.

The original version of this article has been updated.

